# Alcohol-perturbed self-assembly of the tobacco mosaic virus coat protein

**DOI:** 10.3762/bjnano.13.30

**Published:** 2022-04-01

**Authors:** Ismael Abu-Baker, Amy Szuchmacher Blum

**Affiliations:** 1 Department of Chemistry, McGill University, Montréal, Québec, Canada

**Keywords:** alcohol, hydrophobic effect, protein assembly, self-assembly, tobacco mosaic virus

## Abstract

The self-assembly of the tobacco mosaic virus coat protein is significantly altered in alcohol–water mixtures. Alcohol cosolvents stabilize the disk aggregate and prevent the formation of helical rods at low pH. A high alcohol content favours stacked disk assemblies and large rafts, while a low alcohol concentration favours individual disks and short stacks. These effects appear to be caused by the hydrophobicity of the alcohol additive, with isopropyl alcohol having the strongest effect and methanol the weakest. We discuss several effects that may contribute to preventing the protein–protein interactions between disks that are necessary to form helical rods.

## Introduction

Bottom-up fabrication of nanomaterials with precise control over the spatial arrangement of components is of great interest in nanotechnology [1–2]. A promising approach to this issue is the use of templates based on self-assembling biological materials, such as nucleic acids and proteins [3–4]. Biological scaffolds can be programmed through predictable chemical interactions, such as DNA base pairing, disulfide bond formation, and metal coordination, to form complex, well-defined nanostructures [5–6]. Viruses and virus-like particles (VLPs) possess many advantageous properties for biotemplating applications [7–8]. Many viruses and VLPs form monodisperse particles due to the natural capsid protein symmetry and inter-subunit interactions, as well as interactions with encapsidated genetic material [9–10]. Viruses can be obtained in high yields by propagation in host organisms, and viral capsid proteins for VLPs can be obtained through heterologous expression [11–12]. However, working with infectious virus particles poses serious health and environmental safety risks and may require costly containment measures, depending on the virus of interest [13–14]. With this in mind, it may be preferrable to work with virus-like particles composed of the viral capsid proteins without the viral genome.

**Figure 1:**
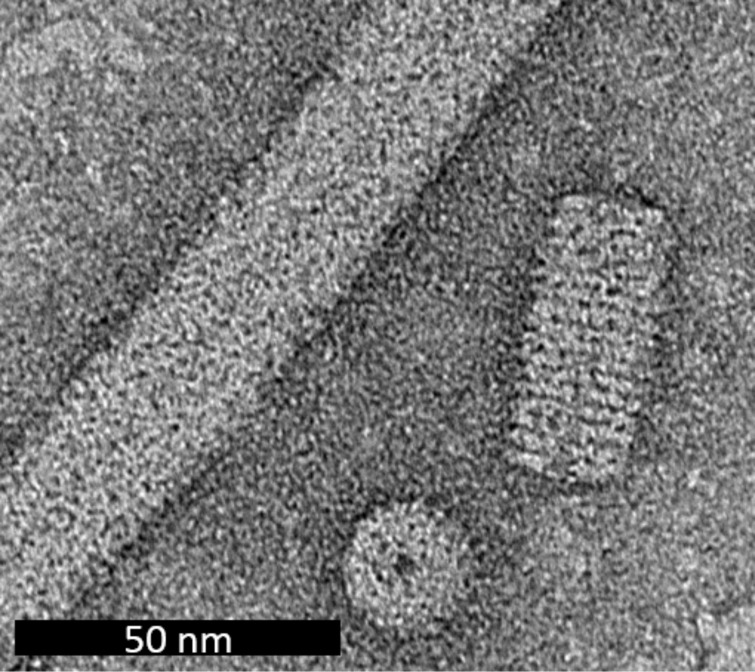
TEM image showing disk, stacked disk, and helical rod particles.

One of the most extensively studied viral templates is the tobacco mosaic virus [15]. The native virus forms helical rod-shaped particles composed of ca. 2130 copies of the coat protein. The particles are 300 nm in length and 18 nm in diameter with a 4 nm central channel. The viral RNA is encapsidated near the inner radius [16]. The tobacco mosaic virus coat protein (TMV-cp) is a 158 amino acid protein with a mass of approximately 17.5 kDa. In the absence of viral RNA, TMV-cp self-assembles into a range of different structures depending mainly on pH and ionic strength (Figure 1). Above neutral pH and at low to moderate ionic strength, the protein exists primarily as a mixture of monomers and small oligomers. This mixture is known as A-protein [17]. Around pH 6.5–7.0, TMV-cp assembles into achiral bilayer disks composed of 17 monomers per layer, 18 nm in diameter with a 4 nm central channel. At high ionic strength and non-acidic pH, these disks can stack on top of each other to form non-helical, rod-like assemblies. There are several known disk aggregates that can be difficult to distinguish between in TEM. The bilayer disk has been reported in two different polymorphs: a polar disk with both layers in the same orientation or a bipolar disk with the two layers related by *C*_2_ symmetry [18–20]. Under basic conditions and at high ionic strength, a four-layer disk aggregate is observed [21]. No distinction is made between these different disk aggregates within the present study. At acidic pH, the disks stack together and rearrange to form long helical rods, retaining the same diameter and central channel [22–23]. The stacked disks and helical rods are distinguishable in transmission electron microscopy (TEM) by the strong transverse striations visible in stacked disks but not helical rods [20,23]. Like many VLPs, helical rod assembly follows a cooperative assembly model, which leads to a bimodal distribution of long rods and small particles (disks and short stacked disks), with few particles at intermediate sizes. While TMV-cp is a promising template for nanomaterials, controlling the multiple assembly states can be challenging, especially when adding additional components with different stability requirements. Apart from adjusting pH and ionic strength, mutating the coat protein has been the main method employed to control TMV-cp self-assembly, with numerous mutants designed to stabilize either the disk or rod forms [24–27]. Herein we describe a simple cosolvent-based method to modify the assembly characteristics of TMV-cp.

The use of dipolar molecules to control the assembly of macromolecular components is well-established with many other systems, including lipids, synthetic polymers, and peptides, but has been the subject of few studies with virus-like particles [28–31]. Lauffer and Shalaby reported that glycine-based molecules promoted the polymerization of TMV A-protein to larger species, likely bilayer disks, as determined by analytical centrifugation and light scattering [32]. They attributed this behaviour to the salting-out effect of glycine and its derivatives. Only the transition from A-protein to disk was investigated, so it is unclear what effect glycine may have on the disk to rod transition. Lee et al. investigated the coagulation of TMV virions in alcohol–water–LiCl solutions [33]. They concluded that ethanol increases hydrophobic interactions between virions, and LiCl screens electrostatic repulsion, leading to significant aggregation of virus particles. The present work focuses on the effect of common alcohols as cosolvents on the TMV-cp assembly from disks to helical rods. Alcohol cosolvents exert a variety of effects on solvent and protein structure. At low concentrations, single alcohol molecules remain dispersed and have a small hydration shell of structured water molecules. As the alcohol content increases, the hydration shells begin to overlap, leading to an extensive hydrogen bonding network and significantly reduced mobility of water molecules. Beyond this point, alcohol molecules begin to cluster together, and eventually, alcohol becomes the bulk phase with small water clusters [34–35]. These changes in solvent structure reduce the solvent permittivity and change solute p*K*_a_ and hydration number [36–37]. Additionally, alcohol–protein interactions can replace protein–protein interactions, altering the protein structure, and even denaturing proteins [38]. In the case of TMV-cp, the presence of low concentrations of alcohol prevents the formation of helical rods when reducing the pH from near neutral to acidic pH, where rods would be expected to form. At higher alcohol concentrations, stacked disks become a major component, with increased hydrophobicity leading to longer stacked disks. The perturbation appears to be based on the hydrophobicity of the cosolvent, with methanol having the weakest effect, and isopropyl alcohol having the strongest effect among the alcohols investigated in this study. This work highlights a simple method to control the self-assembly of virus-like particles without any permanent modifications to the protein structure.

## Results and Discussion

TMV-cp was assembled by dialysis from pH 8.5 to lower pH in the presence of different concentrations of ethanol. Samples were characterized by TEM and dynamic light scattering (DLS). The TMV-cp samples in this work are polydisperse and non-spherical, which complicates the interpretation of DLS data. Because DLS is a light scattering technique, the signal intensity is proportional to the sixth power of the radius [39]. This means that the signal from very small particles can be difficult to detect in the presence of large particles and intensity-average DLS plots appear heavily skewed towards large particles. TMV-cp samples at low pH, which are mixtures of long rods and small disks, are affected by this issue, so in some cases, the disks are not apparent in DLS. For this reason, volume- and number-average DLS results, which are less qualitative but do not favour large particles, are available in Supporting Information File 1. Another complication is that DLS measures the hydrodynamic radius of particles. TMV-cp particles are non-spherical so the particle size from DLS is expected to be significantly lower than the size from TEM, especially for rods. With these issues in mind, DLS in this work should be considered an ensemble qualitative technique to detect the presence of large particles/aggregates.

**Figure 2:**
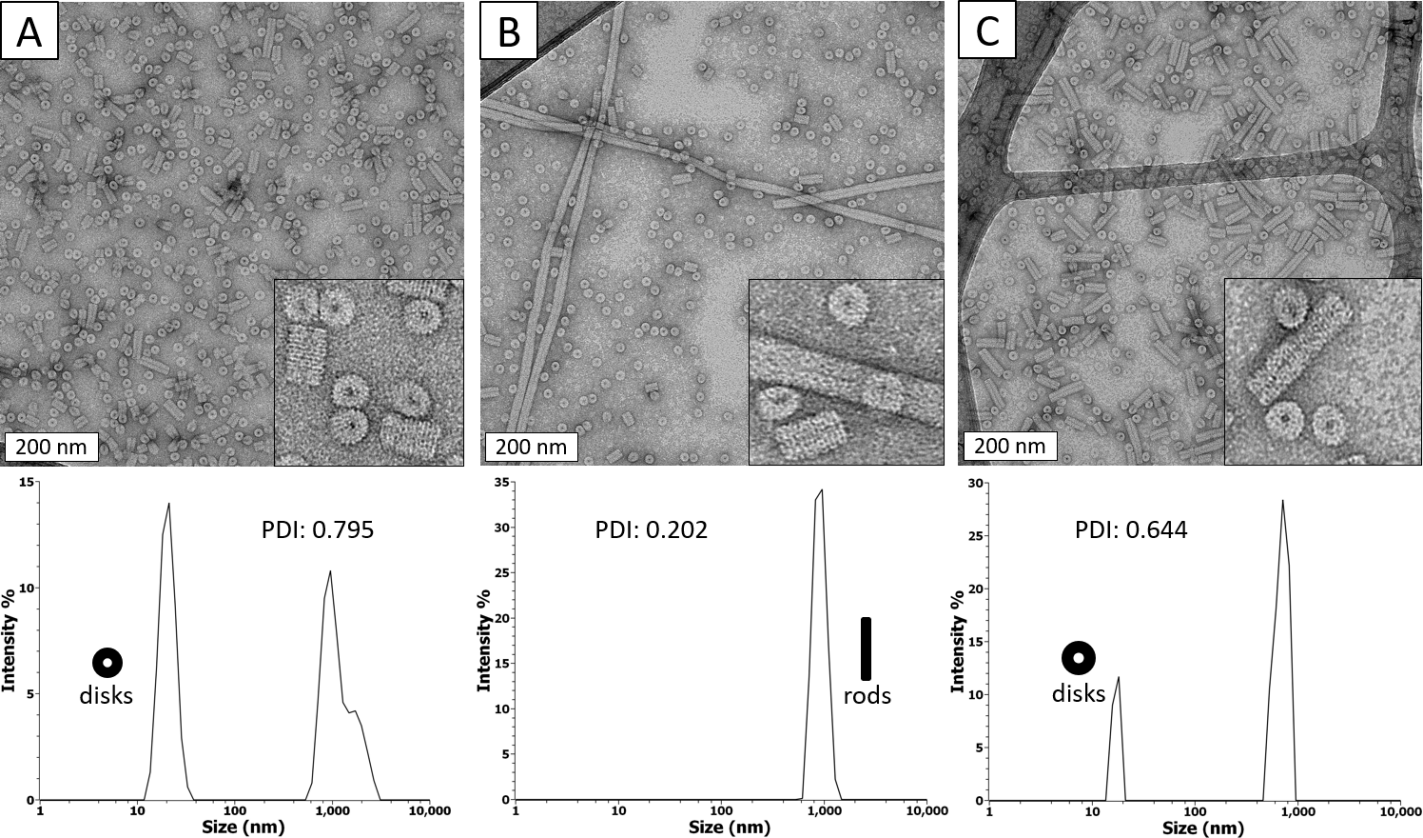
Comparison of TEM images (top) and DLS data (bottom) for TMV-cp assembled under different pH and ethanol concentrations. (A) pH 6.8, no additive; (B) pH 5.5, no additive; (C) pH 5.5, 3.5 mol % ethanol. A lower PDI (polydispersity index) indicates more polydispersity.

**Figure 3:**
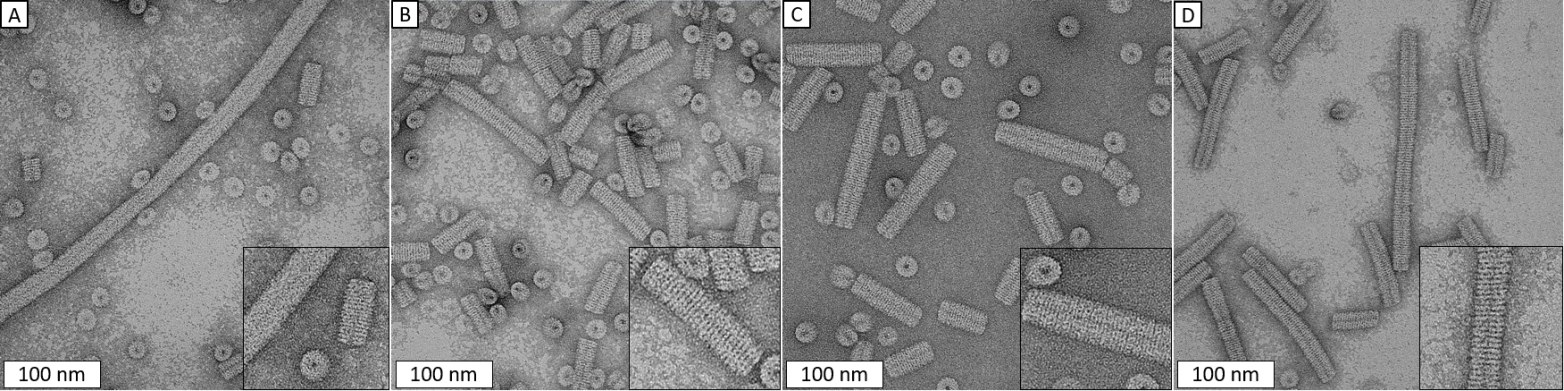
TEM images comparing TMV-cp assembled at pH 5.5 in different concentrations of ethanol. (A) no additive; (B) 3.5 mol % EtOH; (C) 5.0 mol % EtOH; (D) 10.0 mol % EtOH.

The effect of alcohol on TMV-cp assembly was determined by comparing alcohol-containing samples to controls under standard conditions for samples with predominantly disks (pH 6.8) and helical rods (pH 5.5). All samples were characterized after 24 h at room temperature unless otherwise noted. As expected, the pH 6.8 sample showed a mixture of disks and short stacked disks in TEM (Figure 2). DLS showed primarily disks, with a small population of larger particles, which is likely due to dust or aggregation. In addition to disks and stacked disks, long helical rods were observed at pH 5.5 without alcohol present. At pH 5.5, ethanol concentrations below 3.5 mol % showed assemblies of long, helical rods identical to those assembled with no ethanol (Figure S1, Supporting Information File 1). At 3.5 mol % ethanol, helical rods were no longer observed in TEM, and DLS showed a small fraction of larger species, which indicates either minor aggregation or a small population of rods. Instead, disks became the dominant structure, with short stacked disks forming over time. Even after 2 weeks at room temperature, the 3.5 mol % ethanol sample at pH 5.5 was indistinguishable from a pH 6.8 sample with no ethanol (Figure S2, Supporting Information File 1). As shown in Table 1, both the pH 6.8 control and the pH 5 sample with 3.5 mol % ethanol have nearly identical frequency and average length of stacked disks. At pH 5.5, stacked disk assemblies became more common and longer with increasing ethanol concentrations (Table 1 and Figure 3). The 5.0 and 10.0 mol % ethanol samples both showed a significant increase in the length and frequency of stacked disks. 10.0 mol % ethanol caused stacked disks to become the dominant species within 24 h, while 5.0 mol % ethanol showed a transition in the dominant species from disks to stacked disks within 2 weeks. Helical rod assembly was recovered after removal of the ethanol by dialysis in all cases (Figure S3, Supporting Information File 1). These results are consistent with a hydrophobic effect exerted by ethanol. TMV-cp assembly has been shown to be largely driven by hydrophobic effects [40]. It is possible that the hydrophobic alcohol molecules interact favourably with the hydrophobic regions on the faces of TMV-cp disks, thereby preventing the protein–protein contacts necessary for helical-rod formation and stabilizing the disk structure. Disks stack mainly through a solvent network, rather than direct protein–protein interactions, so ethanol does not disrupt the formation of stacked disks [19,21].

**Table 1:** Particle analysis from TEM images. Samples named as “pH, additive (mol %), notes”. NA is no additive. %Disks is the percent of individual disks out of all TMV-cp disks and stacked disks observed. For each sample, at least 1000 particles were analyzed from a single grid. A full statistical evaluation is not possible because only one grid was analyzed per sample. Instead, these results are intended to show trends in particle populations dependent on solution conditions and alcohol content.

Sample	%Disks	Average stacked disk length (nm)	Helical rods

8.5, NA, stock	89.9	23.9	No
7.5, NA	89.7	25.7	No
7.5, 3.5 EtOH	91.7	19.9	No
7.5, 5.0 EtOH	92.4	17.3	No
6.8, NA	88.6	30.3	No
6.8, NA, 2 weeks	63.4	43.8	No
6.8, 3.5 EtOH	82.0	24.1	No
6.8, 5.0 EtOH	92.2	20.8	No
5.5, NA^a^	84.5	23.5	Yes
5.5, NA, 2 weeks^a^	91.1	27.4	Yes
5.5, EtOH removed^a^	90.3	37.0	Yes
5.5, 3.5 EtOH	87.3	31.1	No
5.5, 3.5 EtOH, 2 weeks	68.5	44.8	No
5.5, 5.0 EtOH^b^	62.0	39.5	No
5.5, 5.0 EtOH, 2 weeks^b^	24.4	55.6	No
5.5, 10.0 EtOH^b^	26.9	73.7	No
5.5, 10.0 EtOH, 2 weeks^b^	18.1	93.0	No
5.5, 3.5 MeOH^a^	81.0	28.7	Yes
5.5, 5.0 MeOH^a^	94.8	34.7	Yes
5.5, 10.0 MeOH^b^	45.5	38.7	No
5.5, 3.5 IPA^b^	42.3	35.7	No
5.5, 5.0 IPA^b^	57.1	42.5	No
5.5, 10.0 IPA^b^	65.2	60.7	No

^a^Samples with helical rods; ^b^samples with notable deviation in either %disks or stacked disk length.

**Figure 4:**
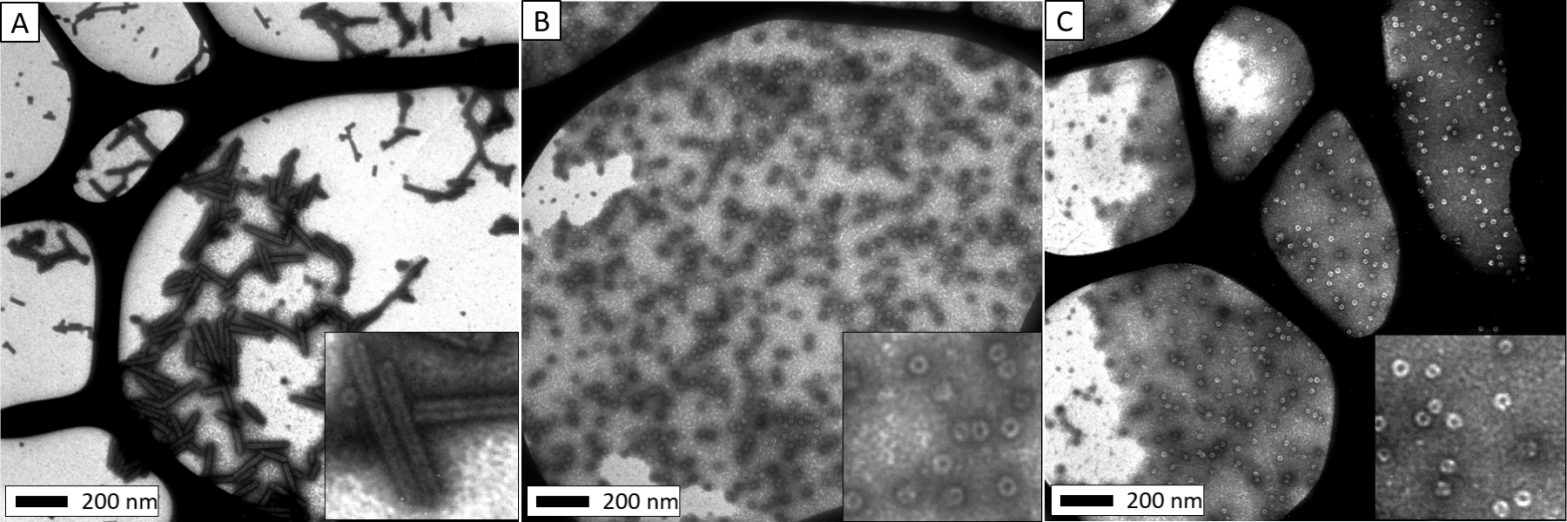
TEM images comparing TMV-cp particles assembled in 5.0 mol % ethanol at different pH values. (A) pH 5.0; (B) pH 6.8; (C) pH 7.5.

The effect of pH on the ethanol-perturbed assembly was also investigated. TMV-cp samples containing 3.5 and 5.0 mol % ethanol were prepared at pH 6.8 and 7.5, and 3.5, 5.0, and 10.0 mol % ethanol samples were prepared at pH 5.0 (Figure 4, Figures S4 and S5, Supporting Information File 1). At pH 6.8 and 7.5, few stacked disks were observed after 24 h. The number and length of stacked disks did increase over time in the pH 6.8 sample, but individual disks remained the dominant species. This is not surprising considering that protonation of Caspar carboxylate pairs around pH 6.5 reduces repulsion between disks, allowing for larger assemblies [41–42]. At higher pH of 6.8 and 7.5, the increased repulsion between subunits may discourage formation of stacked disks. In contrast, at pH 5.0, stacked disks were the dominant species. In 5.0 and 10.0 mol % ethanol, the pH 5.0 samples showed large raft-like clusters of stacked disks (Figure S6, Supporting Information File 1). These clusters could extend for over a micrometre in either the axial or lateral direction. These clusters may be caused by increased hydrophobic interaction strength in the presence of alcohol and reduced particle–particle repulsion near the isoelectric point (5.09) of TMV-cp. This is very similar to what was previously observed in water–alcohol–LiCl solutions [33].

**Figure 5:**
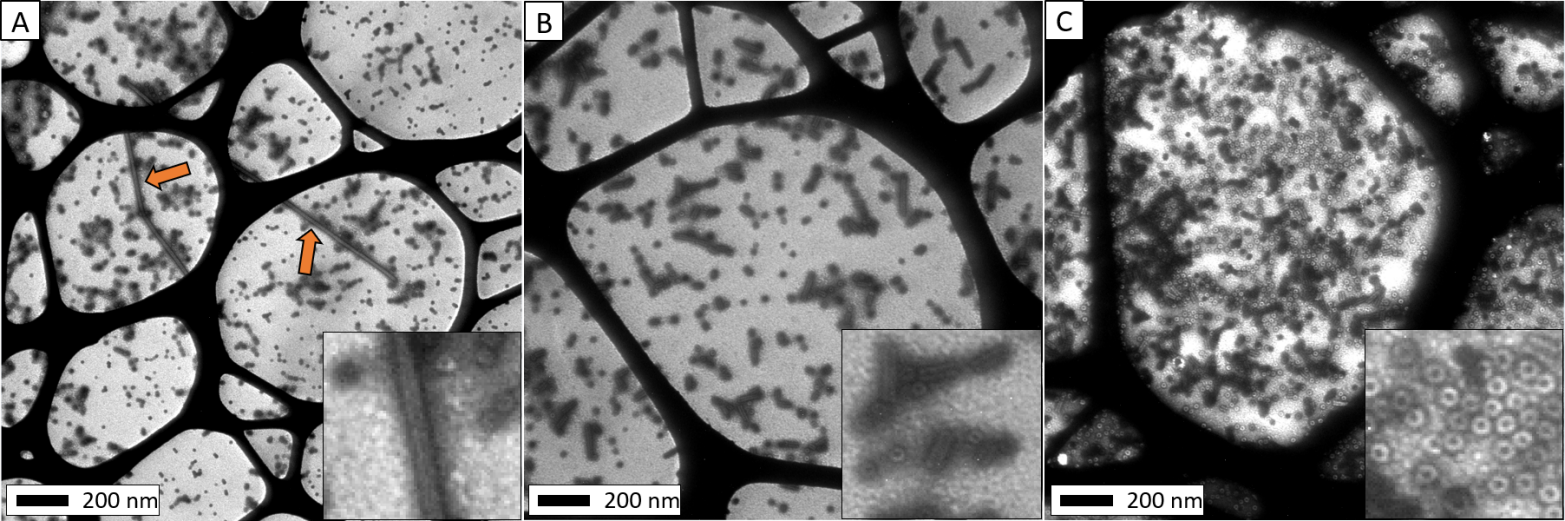
TEM images of TMV-cp assembled at pH 5.5 in the presence of different alcohol additives. (A) 5.0 mol % methanol; (B) 10.0 mol % methanol; (C) 3.5 mol % isopropyl alcohol. Orange arrows indicate long rod species.

To further investigate the effect of alcohols on the protein self-assembly, TMV-cp was assembled by the same procedure at pH 5.5 in the presence of methanol or isopropyl alcohol (Figure 5). As expected, a higher concentration of methanol was required to exert the same effect as ethanol on VLP assembly. 3.5 and 5.0 mol % methanol samples still showed many helical rods, but only disks and stacked disks were observed in 10.0 mol % samples. In contrast, isopropyl alcohol had a very strong effect on TMV-cp assembly, with 3.5 mol % completely eliminating helical rods, and higher concentrations leading to an increase in the average length of stacked disk assemblies (Figure S7, Supporting Information File 1). Stacked disks at high isopropyl alcohol concentrations were longer than at high methanol concentrations and showed large clusters in TEM.

## Conclusion

**Figure 6:**
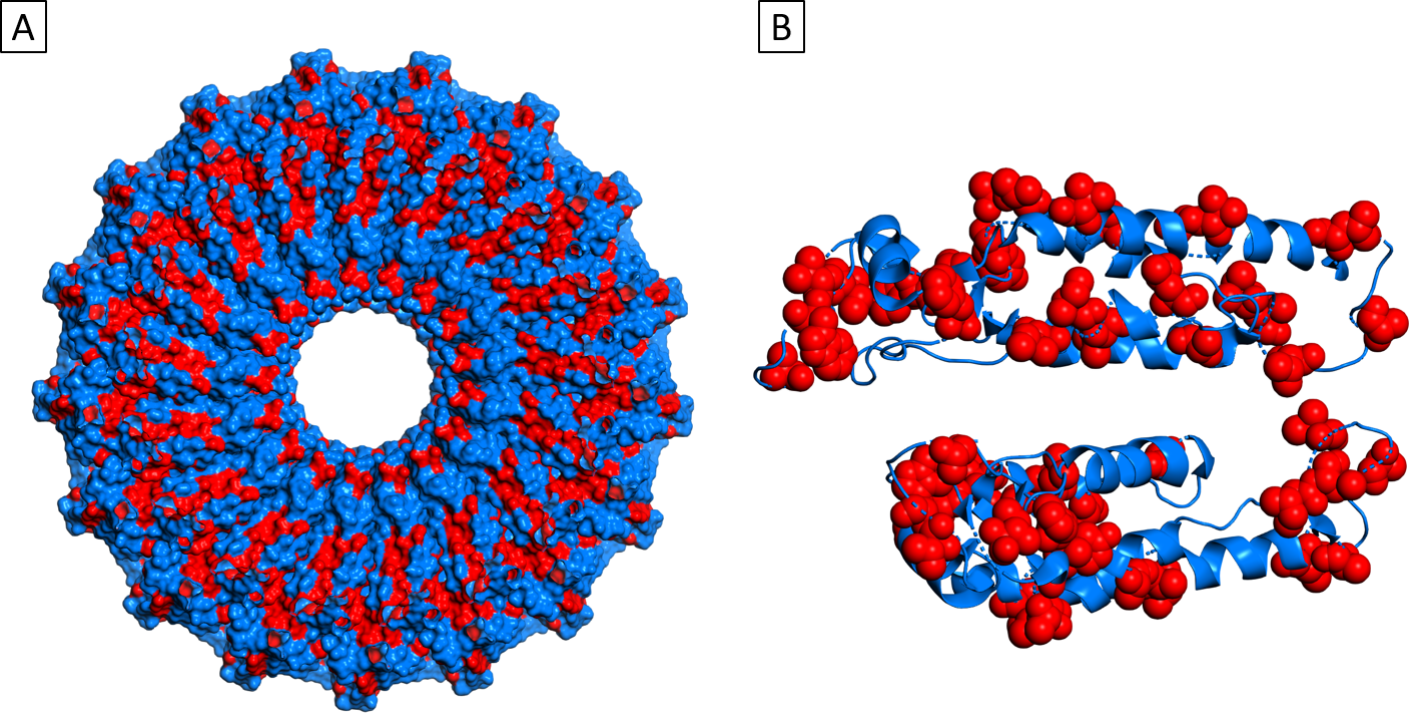
PyMOL [47] schematic showing (A) one face of the disk and (B) the side view of two layers of the disk. Hydrophobic residues are coloured in red. Alcohols may be replacing or strengthening protein–protein interactions between the faces of the disks, preventing helical-rod formation. Based on PDB 1EI7.

The use of dipolar cosolvents to perturb the assembly of TMV-cp has potential applications for nanomaterials. Low concentrations of ethanol or other alcohols can be used to stabilize the disk structure under acidic conditions, where disks would normally assemble into helical rods. This allows for the use of disks under reaction conditions that would normally favour helical rods, or with reactants that are only stable under acidic conditions. As shown in Table 1, increasing the concentration of alcohol favours longer and more frequent achiral stacked disks at acidic pH. In this way, dipolar cosolvents can be used to differentiate between the chiral and achiral rod-shaped particles that TMV-cp forms. There may be several contributing factors to the observed effects. Helical rod assembly is driven by hydrophobic interactions. Thus, alcohol–protein interactions on the face of the TMV-cp disks may replace the protein–protein interactions required for helical-rod formation (Figure 6) [40]. Stacked disks are formed primarily through a solvent network, hence, their formation would not be prevented by alcohol [21]. As was previously shown with glycine, alcohols appear to have a salting-out effect on TMV-cp [32]. However, the increased tendency to aggregate results in more frequent and longer stacked disks rather than helical rods. This could be the result of increased hydrophobic interaction strength, as determined by Lee and co-workers [33]. If disks interact with each other too strongly in the initial non-helical conformation, the energy barrier to rearrange into a helical conformation may become prohibitive. Potential applications for helical and non-helical particles include templated waveguides and negative index materials [43–44]. High alcohol content can also cause aggregation of rod-shaped particles into large raft-like structures, which could allow for templating relatively large surface areas. The effect of alcohol on the TMV-cp assembly was first reported by Bruckman and co-workers. They noted the formation of hexagonally packed sheets of disks when a hexahistidine-tagged TMV-cp (6H-TMV-cp) was dialyzed to pH 5.0 in the presence of 10% ethanol [45]. At the same pH without ethanol, 6H-TMV-cp formed helical rods. However, Bruckman et al. only tested one concentration of ethanol and found that the WT-TMV-cp assembly was unperturbed. With a more extensive investigation, the present work demonstrates that the presence of alcohols in solution has a significant effect on WT-TMV-cp assembly and suggests a mode of action that is relevant to many TMV-cp mutants. Alcohol-perturbed assembly of TMV-cp shows particular promise in combination with mutants that possess additional functionality, as demonstrated by the use of similar hexagonally packed arrays of disks to template sheets of gold nanorings that show promise in plasmonics applications [46]. It is expected that many TMV-cp mutants possessing interesting functionality in the disk or stacked disks phases can have that functionality extended to lower pH by simply including alcohol as a cosolvent.

## Supporting Information

Supporting Information features information on protein expression, purification, and characterization, as well as additional TEM images and DLS data.

File 1 Additional experimental data.
