# Supplementary material for: Alcohol-perturbed self-assembly of the tobacco mosaic virus coat protein
Source: Beilstein J Nanotechnol. 2022 Apr 1;13:355–62. doi: 10.3762/bjnano.13.30 (PMC8978915; doi:10.3762/bjnano.13.30)
Supplement: File 1 — Additional experimental data. [file Beilstein_J_Nanotechnol-13-355-s001.pdf]

BEILSTEIN JOURNAL OF NANOTECHNOLOGY

Supporting Information

for

Alcohol-perturbed self-assembly of the tobacco mosaic virus coat protein

Ismael Abu-Baker and Amy Szuchmacher Blum

Beilstein J. Nanotechnol. **2022,** *13,* 355–362. doi:10.3762/bjnano.13.30

Additional experimental data

License and Terms: This is a supporting information file under the terms of the Creative Commons Attribution License (https://creativecommons.org/licenses/by/4.0). Please note that the reuse, redistribution and reproduction in particular requires that the author(s) and source are credited and that individual graphics may be subject to special legal provisions.
The license is subject to the *Beilstein Journal of Nanotechnology* terms and conditions: (https://www.beilstein-journals.org/bjnano/terms)

Experimental Section

Materials

All chemicals were reagent grade or better. 100% ethanol was purchased from Commercial Alcohols. Potassium phosphate monobasic, potassium phosphate dibasic, 2-propanol, methanol, agar, Coomassie Blue R250, and sodium chloride were purchased from Fisher Scientific. Terrific broth (TB) and lysogeny broth (LB) were purchased from MP Biomedicals. Ampicillin and chloramphenicol were purchased from Research Products International (RPI).

TMV-cp expression and purification

A pET20b vector encoding the sequence of WT-TMV-cp was purchased from NorClone Biotech by reverting an S123C mutant plasmid kindly gifted by Prof. Matthew Francis (UC Berkeley) back to the wild-type sequence. Tuner(DE3)pLysS competent cells (Novagen) were transformed with the vector and streaked on an LB agar plate supplemented with 100 µg/mL ampicillin and 34 µg/mL chloramphenicol. After overnight incubation at 37 °C, a single colony was used to inoculate 10 mL of LB and grown overnight with constant shaking at 37 °C. This saturated growth was used to make frozen glycerol stocks of transformed cells stored at −80 °C. For a typical expression, 20 mL of TB media supplemented with 100 µg/mL ampicillin and 34 µg/mL chloramphenicol was inoculated with a small portion of cells from frozen glycerol stocks and grown overnight at 37 °C with constant shaking at 250 rpm. A 1 mL aliquot of the resulting culture was used to inoculate 1 L of TB, grown for 2.5 h at 37 °C, and then grown overnight at 30 °C. No isopropylthio-β-galactoside (IPTG) was necessary for high levels of protein expression due to a combination of leaky expression of the promoter and compromised cell growth upon induction by IPTG [1,2]. Cultures were harvested by centrifugation and frozen at −80 °C. Frozen cell pellets were thawed, resuspended in lysis buffer (20 mM triethanolamine, 1 mM EDTA, pH 7.4), and lysed by sonication at 50% duty cycle and 60% amplitude. The resulting lysate was clarified by centrifugation at 15,000 rpm for 45 min. The supernatant was collected, and saturated ammonium sulfate solution was added dropwise to a final concentration of 35% (v/v). The precipitate was isolated by centrifugation at 15,000 rpm for 45 min and resuspended in lysis buffer. The resulting solution was dialyzed overnight at 4 °C against the same buffer to remove residual ammonium sulfate. Any remaining precipitate was removed by centrifugation, the solution was diluted, and loaded on a DEAE Sepharose anion exchange column (Cytiva). The protein was eluted using a NaCl gradient of 0–300 mM. Fractions were analyzed by Tris-buffered SDS-PAGE on a 12% gel run in constant voltage mode at 200 V and stained with Coomassie Blue R250. Pure fractions were combined and concentrated. Pure protein was dialyzed into 20 mM potassium phosphate buffer at pH 8.5, concentrated to 2.7 mg/mL, and frozen at −80 °C until further use.

Assembly and characterization of TMV-cp

Stock TMV-cp frozen at 2.7 mg/mL was thawed and diluted to 1.0 mg/mL with a mixture of water and alcohol to obtain the desired alcohol concentration. This solution was dialyzed overnight against the desired buffer, with one buffer change. For pH 5.5 and 5.0, 100 mM sodium acetate buffer was used. 30 mM potassium phosphate buffer was used for pH 6.5 and 7.5. All solutions were adjusted to 100 mM total ionic strength with sodium chloride. Samples were allowed to equilibrate for 24 h after dialysis before characterization. Transmission electron microscopy (TEM) was performed on a 200 kV Talos F200X (Thermo Scientific) and a 5 kV LVEM5 benchtop electron microscope (Delong). Samples were thoroughly mixed by pipette before an 8 µL drop was incubated on an ultrathin carbon on lacey carbon support grid (Ted Pella) for 10 s. The grid was wicked and washed three times with 8 µL drops of MilliQ water before staining. LVEM5 grids were stained for 30 s with a 0.45% uranyl acetate solution. HR-TEM grids were stained for 90 s with 3% uranyl acetate. Dynamic light scattering (DLS) was performed on a Zetasizer Nano ZS (Malvern) at room temperature. DLS data was analysed using Malvern Zetasizer Software. Particle refractive index and absorption were kept at the software's default protein values (1.450 and 0.001, respectively). The viscosity and refractive index of water was used for the solvent as there are negligible changes in those properties within the alcohol concentration range used in this study [3,4]. The correlation function was analyzed using the "general purpose (low resolution)" model from the Malvern software.

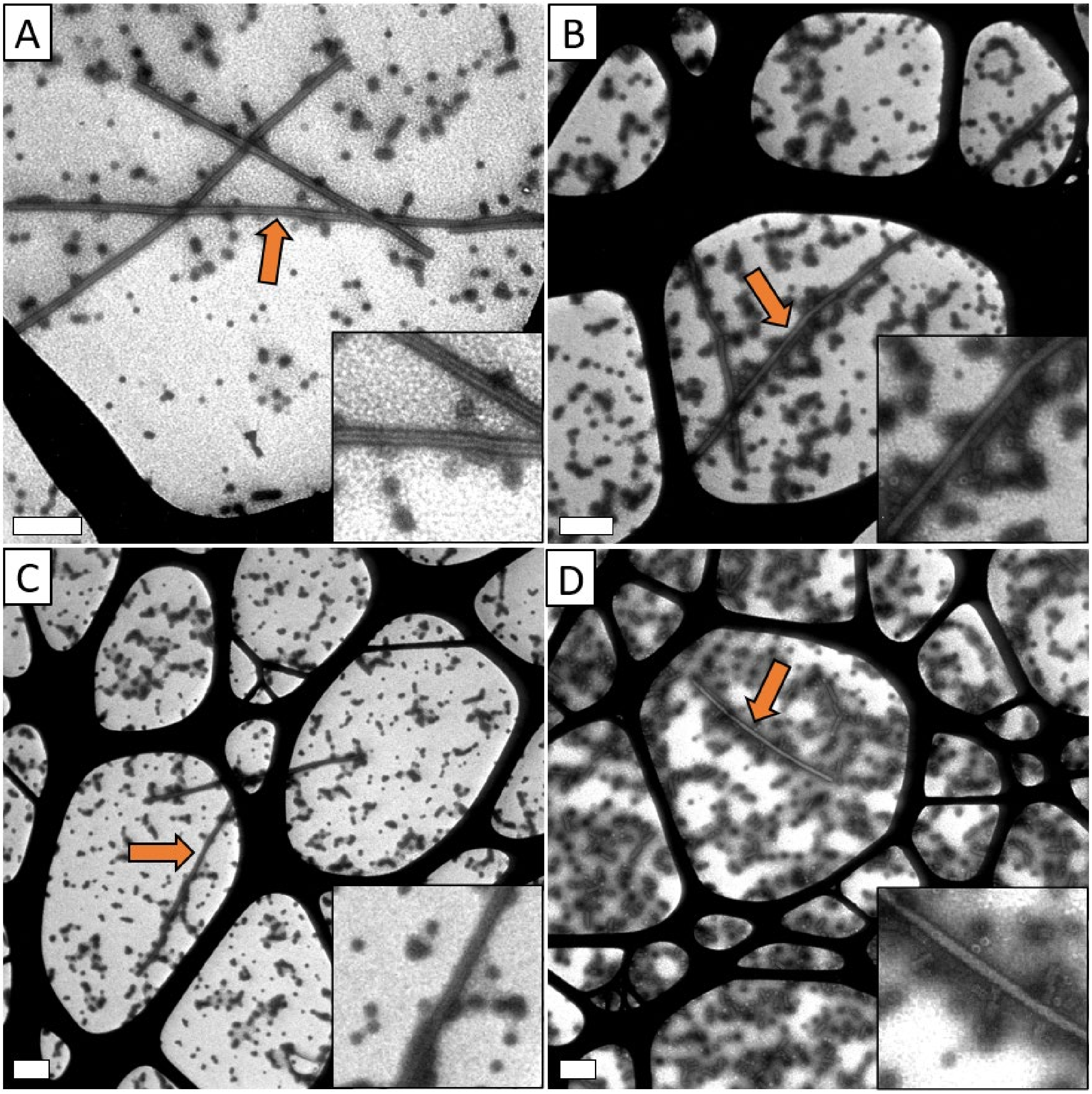

Figure S1: TEM images comparing TMV-cp assembled at pH 5.5 under different concentrations of ethanol. (A) 1.0 mol % EtOH; (B) 2.0 mol % EtOH; (C) 2.5 mol % EtOH; (D) 3.0 mol % EtOH. Scale bars are 200 nm. Orange arrows indicate helical rods.

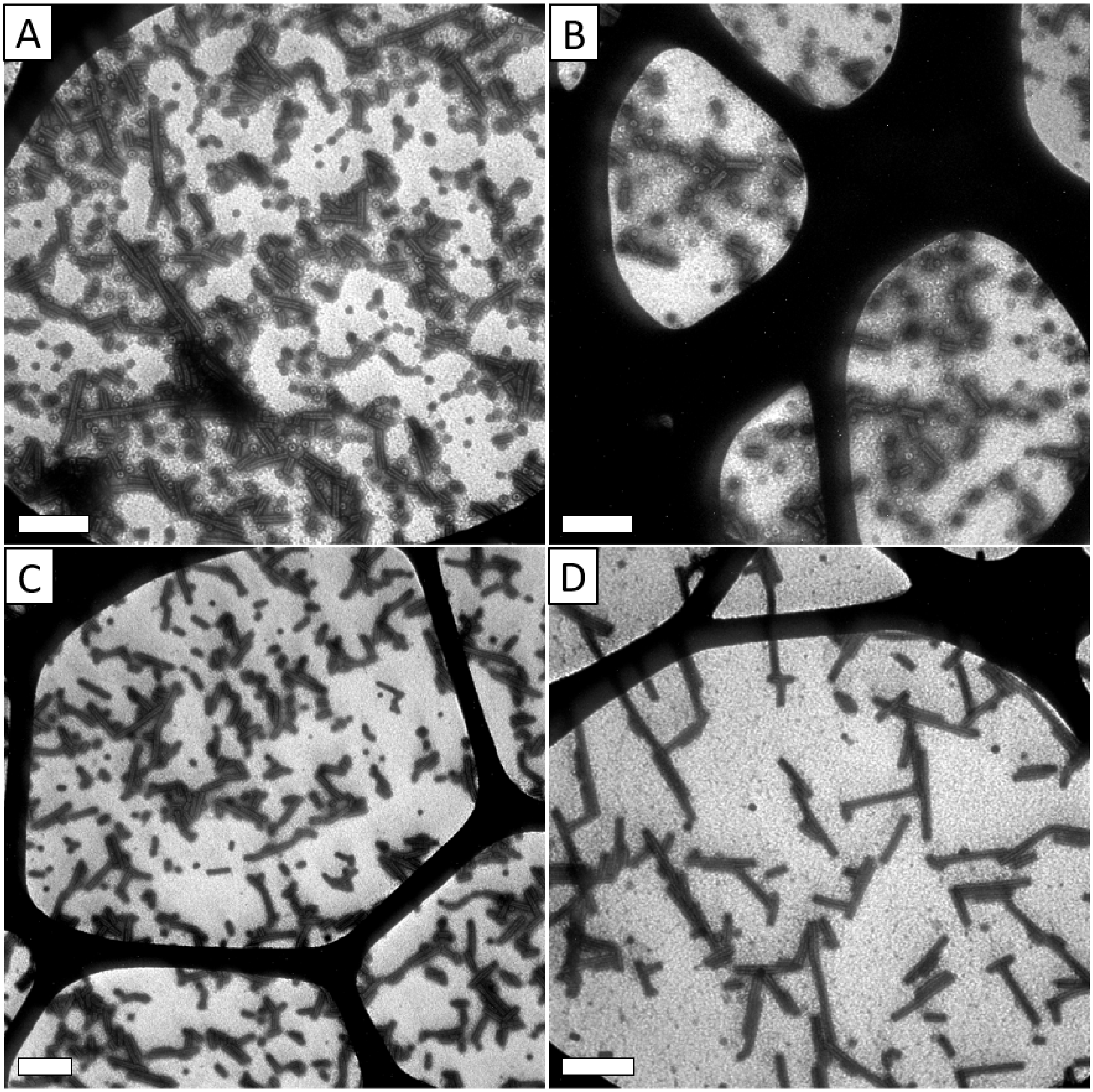

Figure S2: TEM images of samples after 2 weeks at room temperature. (A) pH 6.8, no additive; (B) pH 5.5, 3.5 mol % EtOH; (C) pH 5.5, 5.0 mol % EtOH; (D) pH 5.5, 10.0 mol % EtOH. Scale bars are 200 nm.

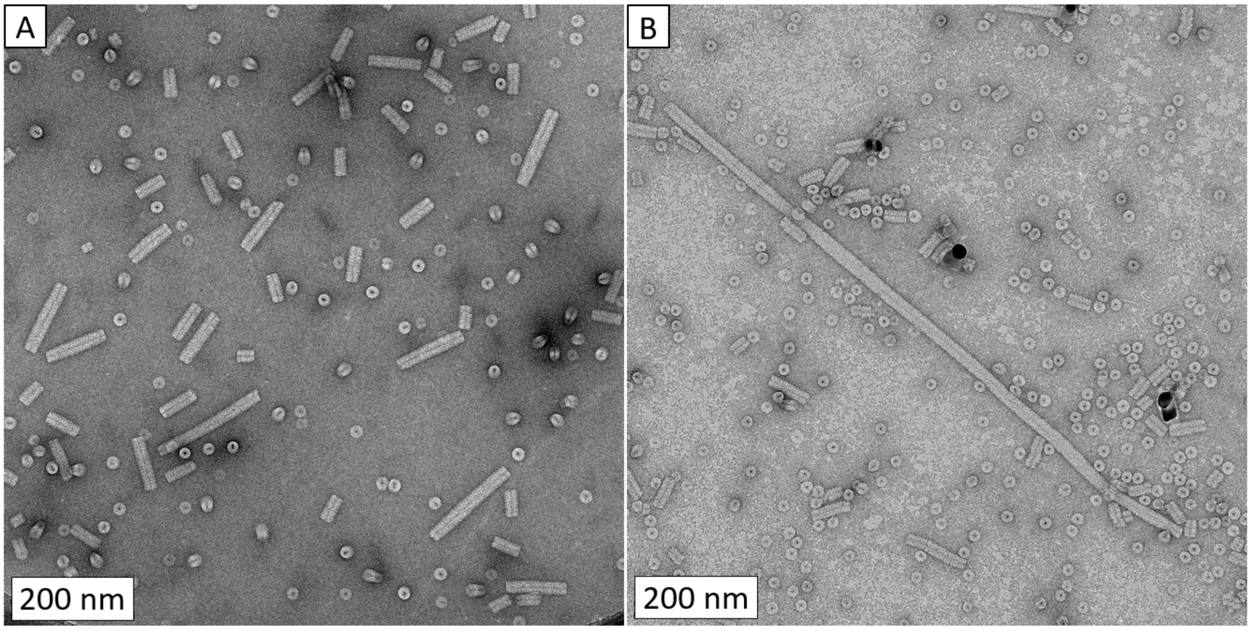

Figure S3: TEM images of TMV-cp at pH 5.5. (A) With 3.5 mol % ethanol; (B) same sample after removal of ethanol by dialysis.

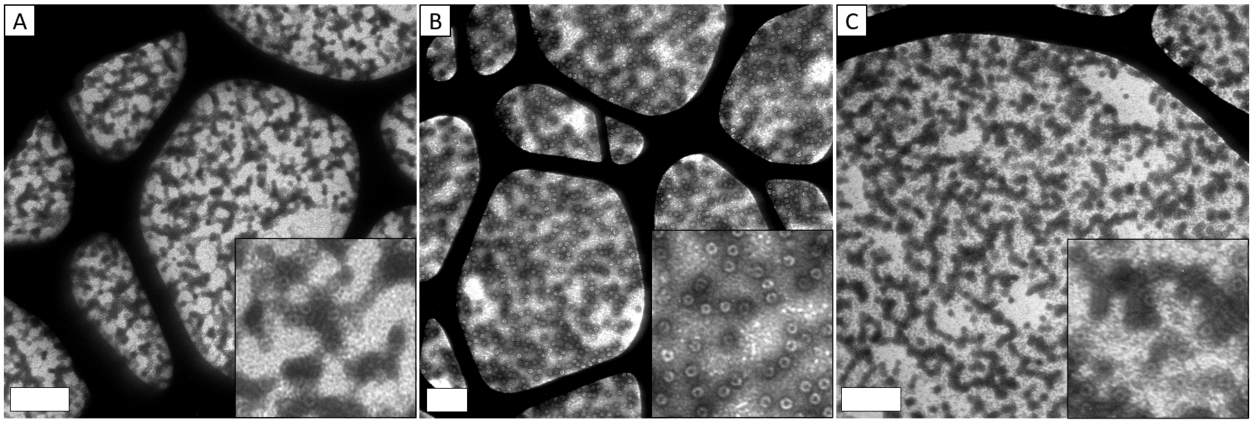

Figure S4: TEM images of TMV-cp at different pH values and ethanol concentrations. A) pH 7.5, 0.0 mol % EtOH, B) pH 7.5, 3.5 mol % EtOH, C) pH 6.8, 3.5 mol % EtOH. Scale bars are 200 nm.

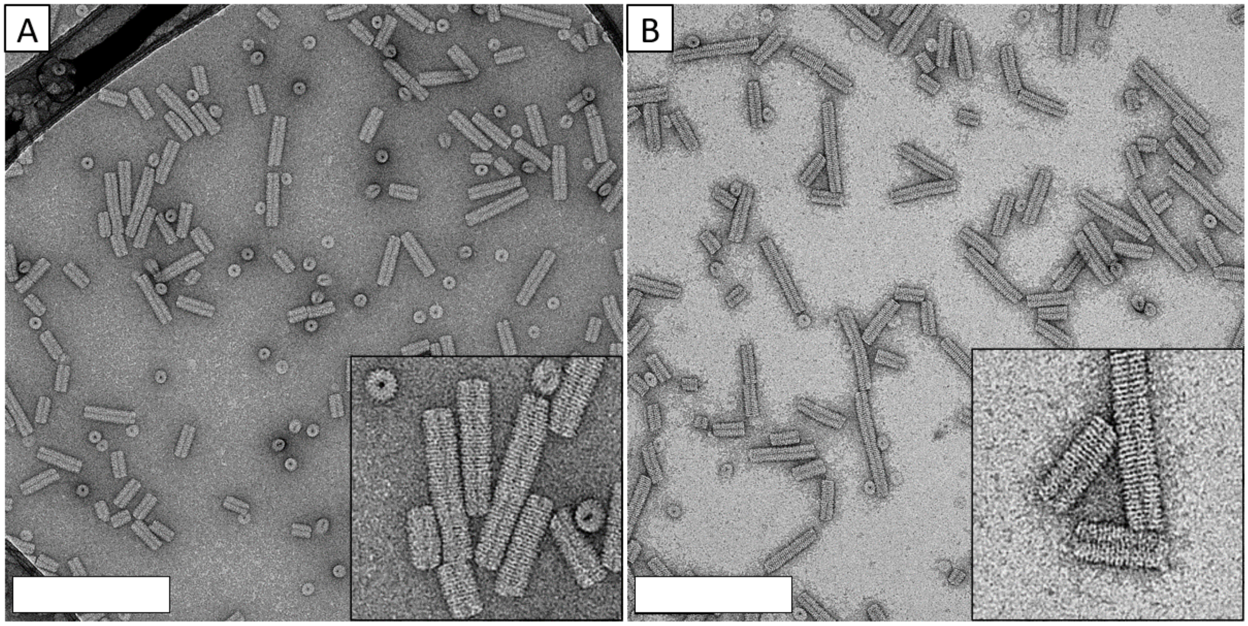

Figure S5: TEM images of TMV-cp at pH 5.0 in different ethanol concentrations. (A) 5.0 mol % EtOH; (B) 10.0 mol % EtOH. Scale bars are 200 nm.

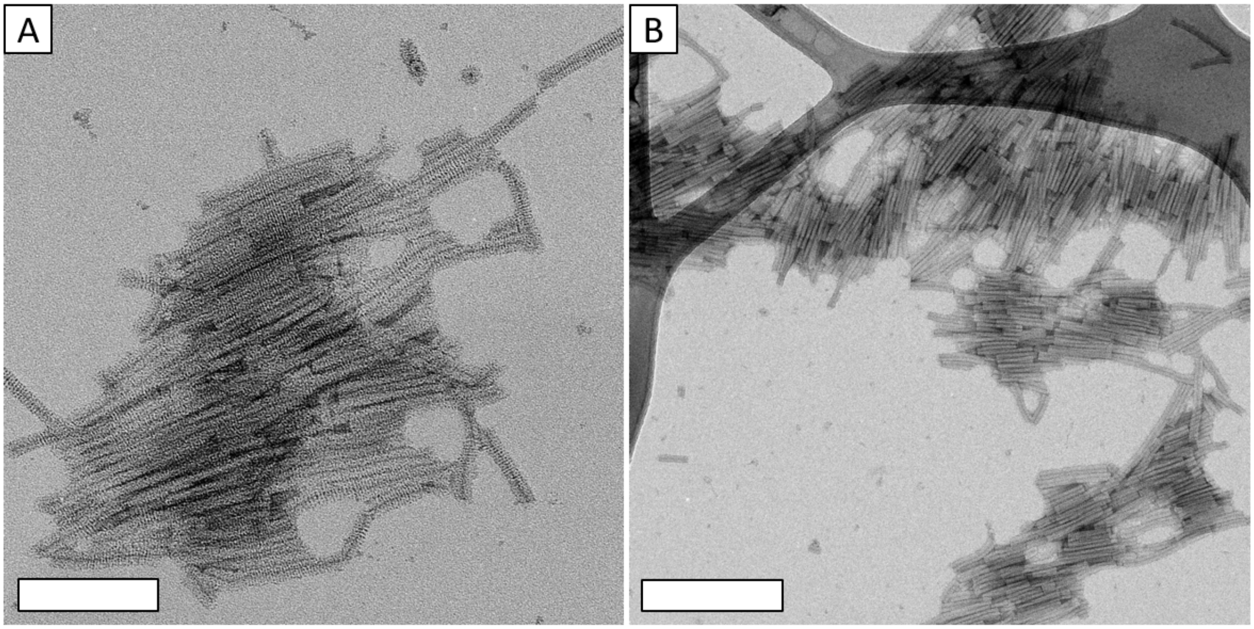

Figure S6: Representative TEM images of TMV-cp showing large raft-like structures at pH 5.0 with 10.0 mol % ethanol.

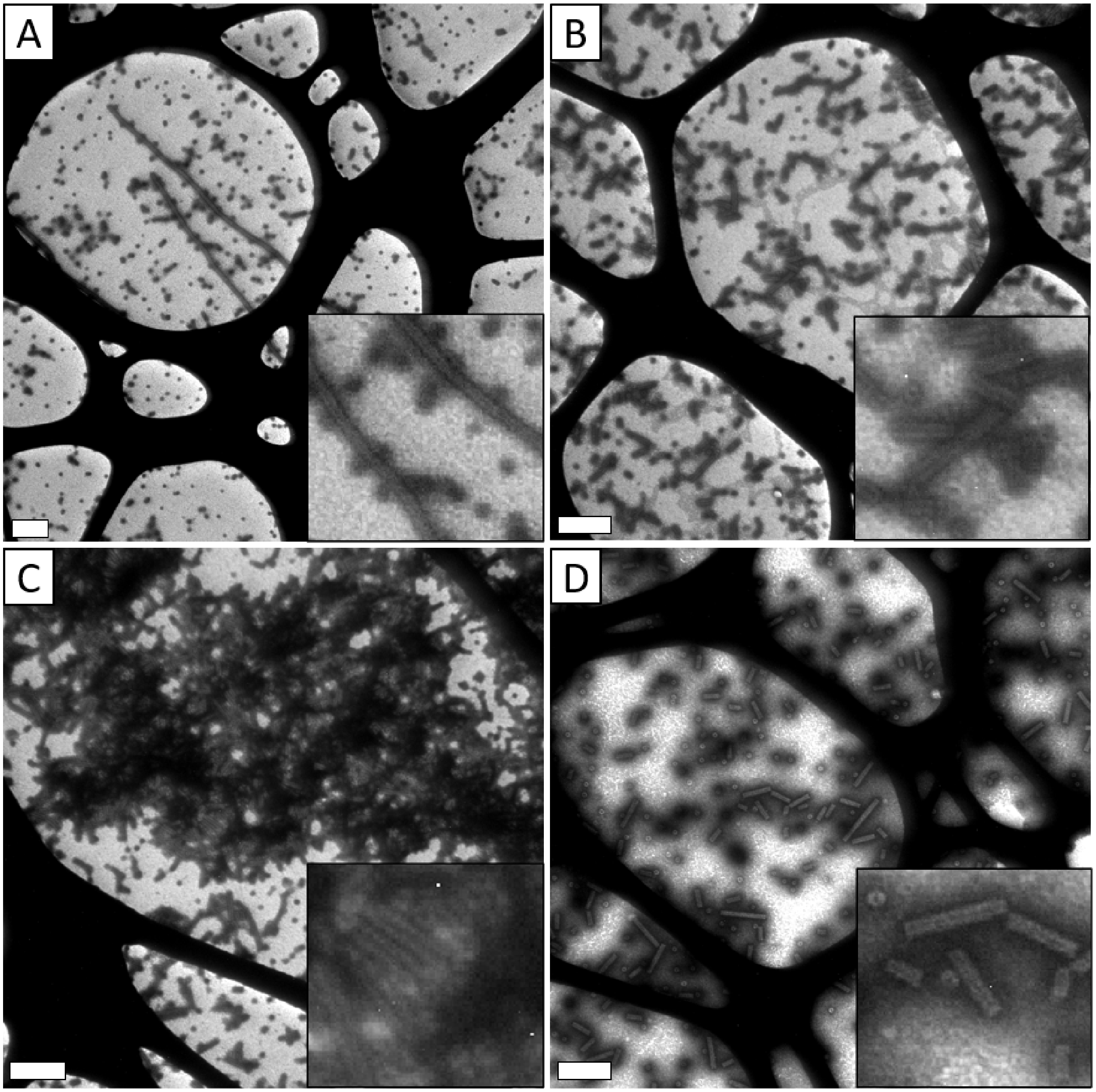

Figure S7: TEM images of TMV-cp at pH 5.5 assembled in different concentrations of methanol or isopropyl alcohol. (A) 3.5 mol % methanol; (B) 5.0 mol % isopropyl alcohol, (C) 5.0 mol % isopropyl alcohol showing clustering; (D) 10.0 mol % isopropyl alcohol. Scale bars are 200 nm.

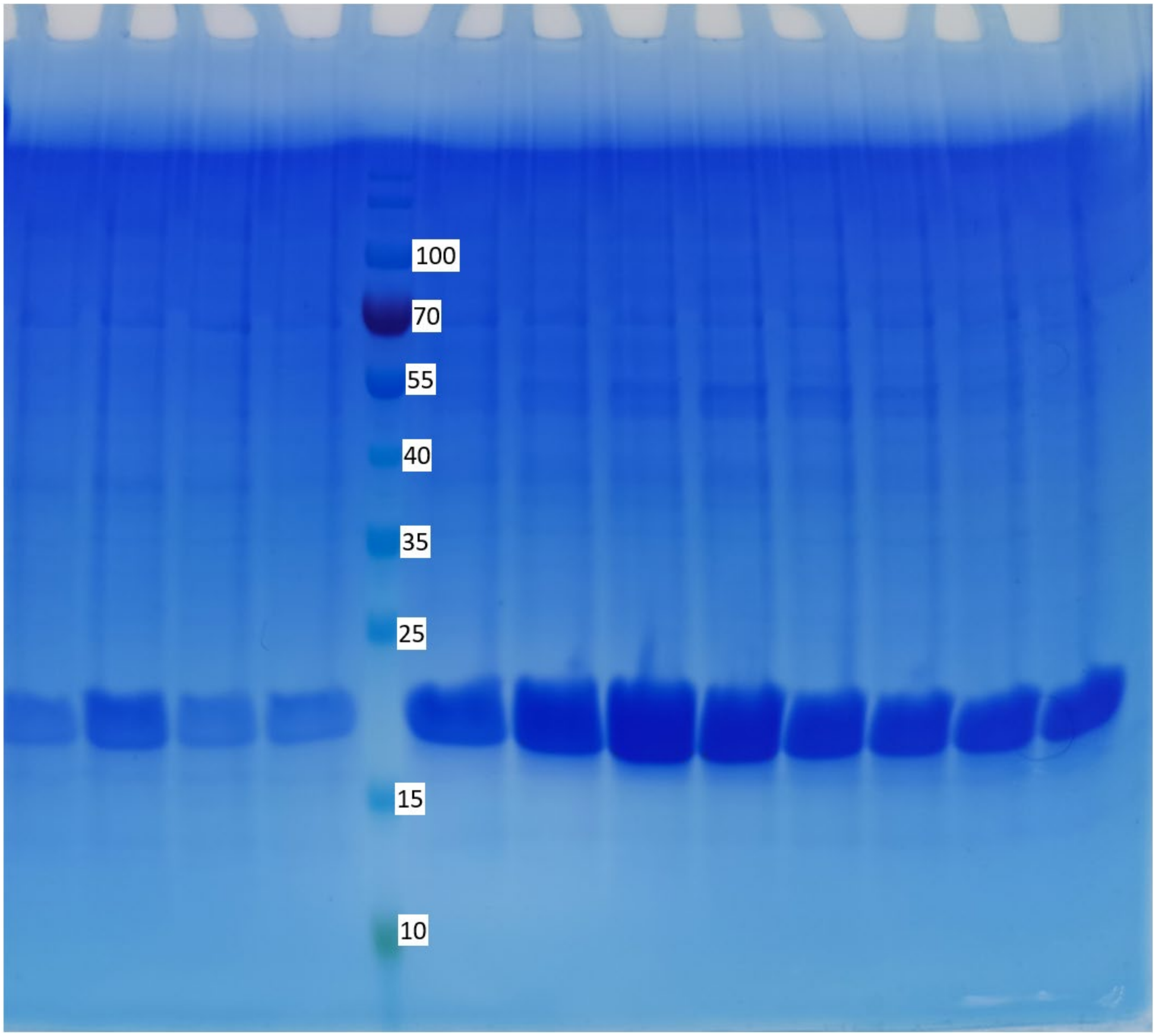

Figure S8: SDS-PAGE gel of eluted fractions from a typical WT-TMV-cp purification. The target protein shows heavy bands at ca. 17.5 kDa.

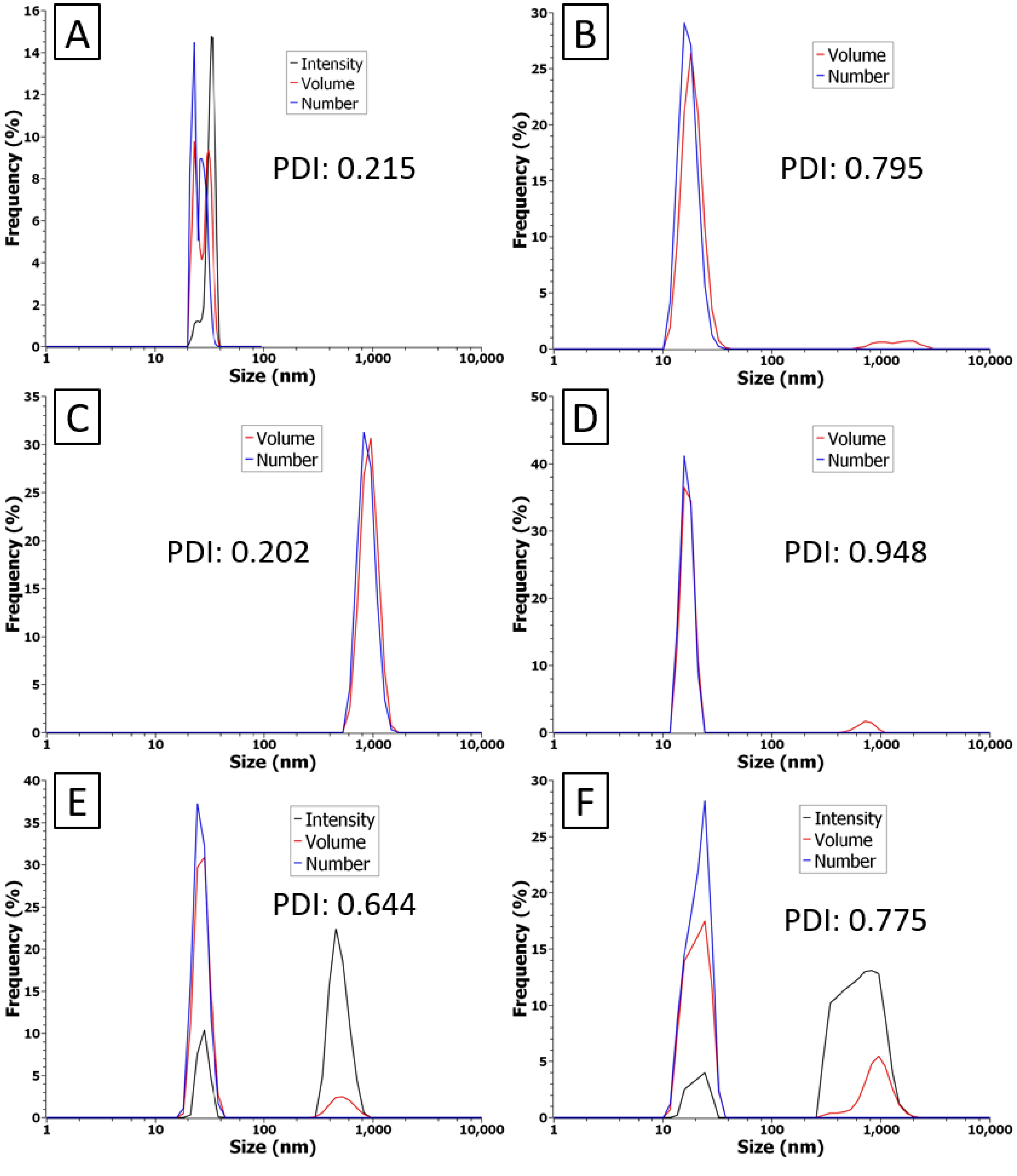

Figure S9: DLS data of TMV-cp assembled under different conditions. (A) pH 8.5 stock solution; (B) pH 6.8, no additive; (C) pH 5.5, no additive; (D) pH 5.5, 3.5 mol % EtOH; (E) pH 5.5, 5.0 mol % EtOH; (F) pH 5.5, 10.0 mol % EtOH. Line colour indicates averaging type as follows: black – intensity, red – volume, blue – number.

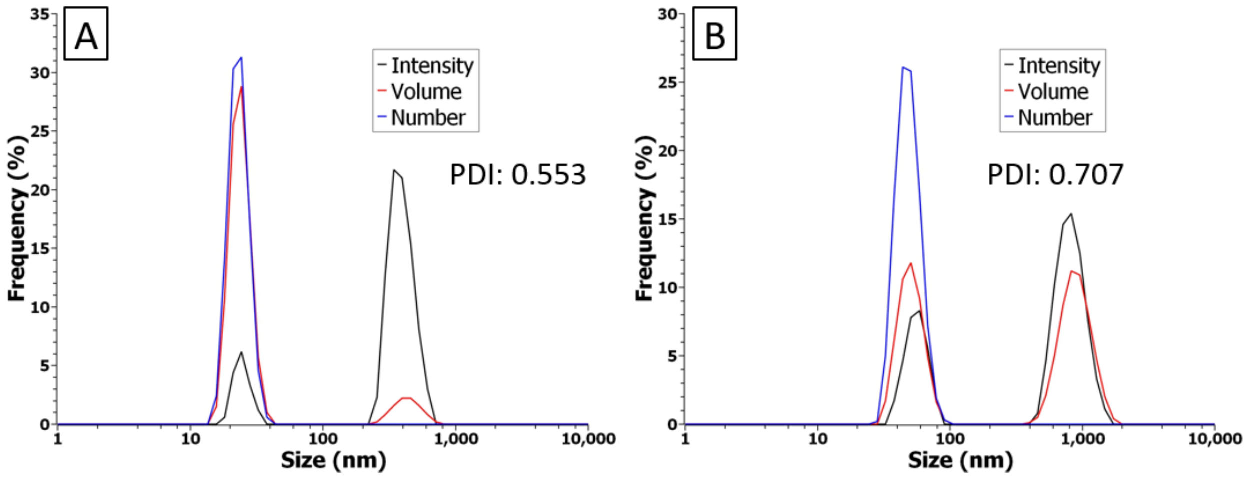

Figure S10: DLS data of TMV-cp at pH 5.5 assembled in different alcohols. (A) 3.5 mol % MeOH; (B) 3.5 mol % IPA. Line colour indicates averaging type as follows: black – intensity, red – volume, blue – number.

References

1. Dubendorf, J. W.; Studier, F. W. *J. Mol. Biol.* **1991,** *219,* 45–59. doi:10.1016/0022-2836(91)90856-2
2. Kadri, A.; Wege, C.; Jeske, H. *J. Virol. Methods* **2013,** *189,* 328–340. doi:10.1016/j.jviromet.2013.02.017
3. Scott, T. A., Jr. *J. Phys. Chem.* **1946,** *50,* 406–412. doi:10.1021/j150449a003
4. Tanaka, Y.; Yamamoto, T.; Satomi, Y.; Kubota, H.; Makita, T. *Rev. Phys. Chem. Jpn.* **1977,** *47,* 12–24.
